# Characterization of a deformable beating cardiac phantom with real‐time dosimetric capabilities for validation of MRI‐guided heart radiotherapy

**DOI:** 10.1002/mp.70313

**Published:** 2026-02-12

**Authors:** Manon M. N. Aubert, Prescilla Uijtewaal, Kalin I. Penev, Yoan LeChasseur, Nick Hartman, Laurie J. M. de Vries, Paris Tzitzimpasis, Pim T. S. Borman, Stefano Mandija, Martin F. Fast, Astrid L. H. M. W. van Lier

**Affiliations:** ^1^ Department of Radiotherapy University Medical Center Utrecht Utrecht The Netherlands; ^2^ IBA QUASAR London Ontario Canada; ^3^ Medscint Québec City Quebec Canada; ^4^ Computational Imaging Group for MR Diagnostics & Therapy University Medical Center Utrecht Utrecht The Netherlands

**Keywords:** deformable heart model, intra‐fraction motion management, MRI‐guidance, real‐time dosimetry

## Abstract

**Background:**

Stereotactic arrhythmia radioablation (STAR) has emerged as a salvage treatment for patients with ventricular tachycardia, and the MR‐linacs offer MRI‐guidance during such treatment. However, available workflows on MR‐linacs are not yet optimized and characterized dedicatedly to perform heart radiotherapy due to a lack of realistic MRI‐compatible cardiac phantoms suitable for real‐time dosimetry.

**Purpose:**

This work introduces a newly designed, deformable, and MRI‐compatible cardiac phantom with real‐time and multi‐point dosimetric capabilities, characterizes its MRI properties and mechanical behavior, and showcases its use for MR‐linac end‐to‐end workflow testing.

**Methods:**

This cardiac phantom (IBA QUASAR, London, Ontario, Canada) is composed of a deformable heart model — representing the left and right ventricles — with integrated Plastic Scintillation Detectors (Medscint, Quebec, Quebec, Canada) (PSDs), a section filled with contrast solution, and a piston compatible with the motor of the QUASAR^TM^ MRI^4D^ motion phantom. We determined its T1 and T2 values by acquiring gold standard inversion recovery and spin echo sequences. We evaluated its 2D deformation and mechanical behavior by performing an MRI‐based analysis inspired by dynamic mechanical analysis. We evaluated its 3D deformation and mechanical behavior by acquiring 3D scans at different motor positions, and by applying deformable image registration. We evaluated the reliability of the PSD measurements by testing the repeatability, linearity, and dose rate dependency of their measured dose on a 1.5 T Unity MR‐linac (Elekta AB, Stockholm, Sweden). We evaluated the dosimetric impact of motion and motion mitigation on the dose measured by the PSDs by delivering a STAR plan in different cases: static, motion (cardiorespiratory or cardiac motion), and motion with gating (MR‐linac clinical workflow).

**Results:**

On average, T1 values were T1_constrast_solution_ = 1130 ± 44 ms, T1_left_ventricle's_wall_ = 763 ± 76 ms, and T1_right_ventricle's_wall_ = 775 ± 69 ms, and T2 values were T2_constrast_solution_ = 155 ± 16 ms, T2_left_ventricle's_wall_ = 49 ± 12 ms, and T2_right_ventricle's_wall_ = 185 ± 35 ms. For the 2D deformation, no phase lag of deformation (i.e., ventricular area) with respect to the piston motion and no hysteresis behavior were observed. For the 2D and 3D deformation, linear relationships between piston positions and ventricular areas or volumes of interest were observed (*R* ^2^ = 0.99 and *R* ^2^ = 1.0, respectively). For the 3D deformation, negligible differences between volumes before and after PSDs integration were obtained (largest absolute percentage difference of 1.1%). The maximum deformation of the heart model was 5 mm. For the dosimetric capabilities, the reliability of the PSD measurements was shown (repeatability: coefficient of variation ≤0.31%, linearity: *R* ^2^ = 1.0, dose rate dependency: coefficient of variation ≤0.72%). Dose rates measured by the PSDs over time during STAR delivery were fluctuating in phase with the motion pattern. Percentage errors on cumulative doses with respect to static cases when cardiorespiratory or cardiac motion were applied were, respectively, up to −28.6% and −0.7% in motion cases, and up to −3.1% and 0.8% in gating cases.

**Conclusions:**

This deformable and MRI‐compatible cardiac phantom has good MRI contrast, and its heart model exhibits elastic behavior. Its integrated PSDs offer real‐time and multi‐point dose measurements, and their reliability was demonstrated. We successfully showed that this cardiac phantom enables end‐to‐end testing for heart radiotherapy on MR‐linacs.

## 1 INTRODUCTION

Stereotactic body radiation therapy (SBRT)^1^ is increasingly used in thoracic radiotherapy to treat lung cancers^2^, ^3^ and targets localized in the heart. SBRT can be used in heart radiotherapy to treat malignant cardiac tumors, but it is also used as a salvage treatment for ventricular tachycardia (VT). In malignant cardiac tumors, conventional radiotherapy is often used as an adjuvant therapy to improve patient prognosis.^4^, ^5^ However, SBRT is an encouraging alternative to conventional radiotherapy.^6^, ^7^, ^8^ In VT, most patients have structural heart disease^9^, ^10^ and their VT are mainly due to scar‐mediated reentries (local reentrant circuits that can interfere with the normal electrical activity of the heart). These patients are usually prescribed an implantable cardioverter‐defibrillator (ICD) to prevent the risk of sudden cardiac death. However, this cardiac device does not prevent VT episodes, and different approaches are used to reduce the VT burden. When the VT cannot be managed with the standard approaches, patients are eligible for a SBRT procedure (referred to as stereotactic arrythmia radioablation (STAR)).

Although SBRT has shown potential in thoracic and heart radiotherapy^2^, ^3^, ^11^, ^12^, ^13^, this procedure has not been fully embraced due to the risk of radiation‐induced (cardiac) toxicity. SBRT can only be used if the target and its surrounding tissues are sufficiently localized to accurately receive the planned dose. The heart is, however, a complex tissue structure that contracts periodically (frequency = [50–100] bpm)^14^ to maintain the blood flow in the body and it changes position periodically (frequency = [12–19] bpm)^14^ as the lungs ventilate. As a consequence, the cardiac substructures are displaced due to cardiorespiratory‐induced motion and the amplitude of motion is substructure and patient‐dependent.^15^ To reduce the risk of radiation‐induced toxicity, this motion needs to be resolved by image‐guidance during treatment.

Magnetic resonance imaging (MRI) provides a superior soft‐tissue contrast, which allows to better visualize cardiac function, anatomy, and motion.^10^, ^16^ As a consequence, the MRI‐guidance offered by hybrid MRI–radiotherapy linear accelerator (MR‐linac) systems is attractive to increase geometric and dosimetric accuracy. Cancers, as well as scar‐mediated VT, have already been treated on MR‐linacs and results are encouraging.^17^, ^18^, ^19^, ^20^ However, available workflows on MR‐linacs are not yet optimized and characterized dedicatedly to perform heart radiotherapy: workflow development and quality assurance still need to be accomplished to fully exploit MRI‐guidance advantages.

Workflows for cardiac applications on MR‐linacs should ideally be developed and validated by using a cardiac phantom that is suitable for end‐to‐end testing. However, such phantom needs to meet specific requirements. First, to ensure spatial accuracy and realistic testing, this cardiac phantom should sufficiently mimic the cardiac structure, properties, and motion. The surrounding environment should also mimic the patient conditions (e.g., proximity of the lungs — air‐filled, most likely presence of an ICD). Second, to ensure dosimetric accuracy, this cardiac phantom would ideally enable real‐time and multi‐point dose measurements. It would allow to follow the dose delivered over time and at different positions (e.g., to mimic target site and organs at risk). Lastly, to be usable on an MR‐linac, this cardiac phantom should be MRI‐compatible.

To the best of our knowledge, the existing thoracic motion phantoms do not yet meet all of these requirements. While some of them are MRI‐compatible (Model DHP‐MRI, Shelley Medical Imaging Technologies, Canada or Adult Heart Phantom, TruePhantom Solutions, Canada), they do not enable dosimetric testing. Others enable dosimetric testing (Model 008C‐01, Sun Nuclear, USA), but are not MRI‐compatible. A novel cardiac phantom was recently developed at the University of Wisconsin–Madison^21^ and opens new possibilities: it is MRI‐compatible, has a realistic cardiac structure, and enables dosimetric testing. However, it also has limitations as its nondeformability. Concurrently, a 3D‐printing‐based method has been developed to create deformable 3D dosimeters in anthropomorphic phantoms.^22^ However, they do not enable real‐time dose measurements, which limit their potential for end‐to‐end testing.

Here we introduce a newly designed cardiac motion phantom to help workflow developments for cardiac applications on MR‐linacs. This cardiac phantom is deformable, MRI‐compatible, and enables real‐time and multi‐point dose measurements. In this work, we characterize its static and dynamic properties and behavior, and we showcase its use for MR‐linac end‐to‐end workflow testing.

## 2 MATERIALS AND METHODS

We divided our work into two main parts to evaluate the characteristics of the phantom and its capabilities to perform accurate real‐time and multi‐point dose measurements during a STAR plan delivery. In the first part, we focused on its MRI properties (T1 and T2 values) and we compared those to in vivo properties (Section 2.2.1). We then focused on its 2D deformation and mechanical behavior (Section 2.2.2). We finally focused on its 3D deformation and mechanical behavior, and its stroke volume (Section 2.2.3). Elastic and viscous behaviors were of interest to evaluate the mechanical behavior of the phantom. In the second part, we focused on the creation of our STAR plan (Section 2.3.1). We then focused on the reliability of the dosimeter measurements (Section 2.3.2). We finally focused on the dosimetric impact of motion and motion mitigation (using gating) on the dose measured by the dosimeters (Section 2.3.3).

### 2.1 Phantom design and setup

**FIGURE 1:**
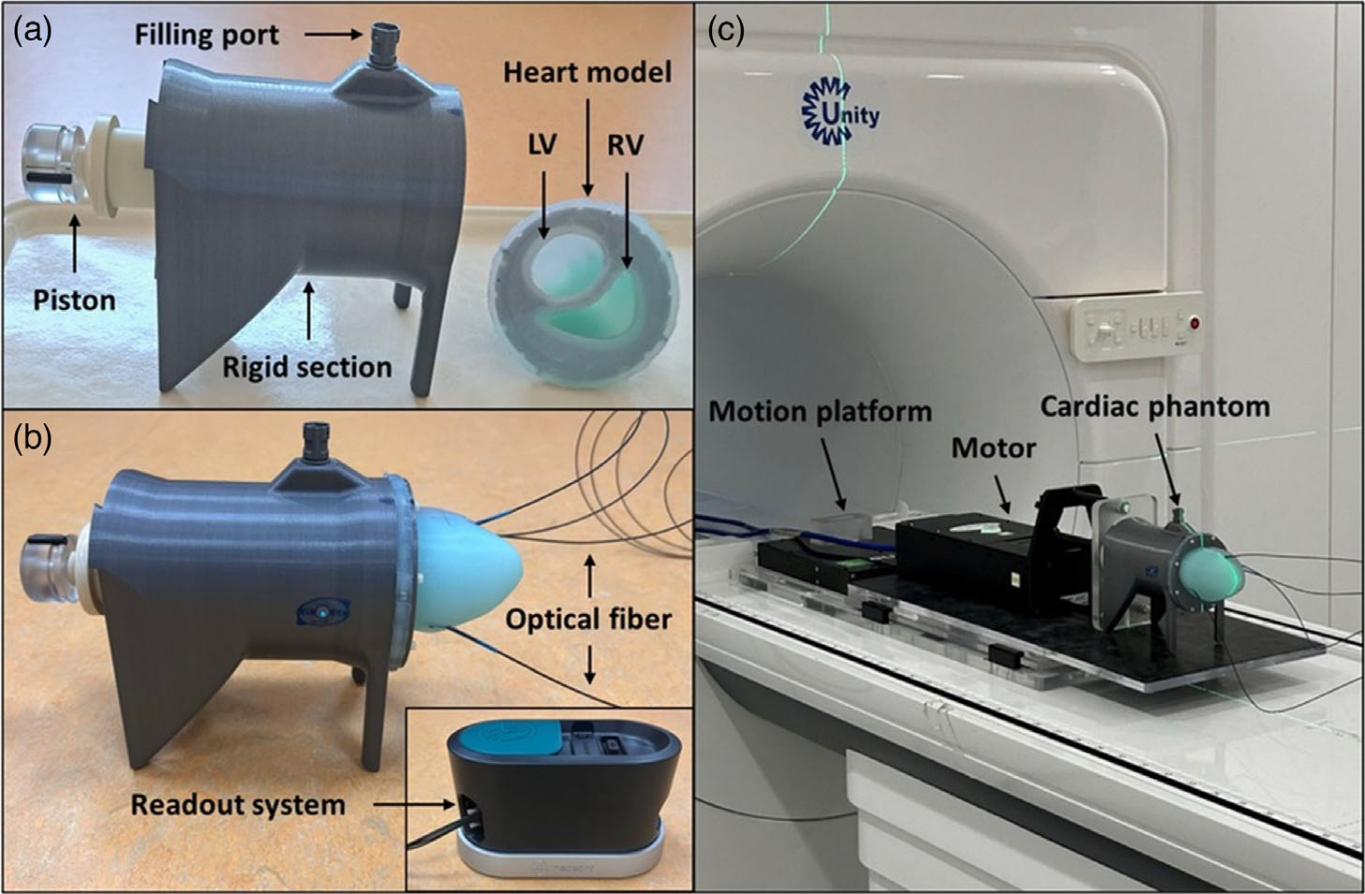
Cardiac phantom design and setup. (a) Opened assembly. (b) Cardiac phantom with integrated PSDs and readout system. (c) Setup at the 1.5 T Unity MR‐linac. The cardiac phantom is connected to the motor of the motion phantom. Here, both are placed on top of the motion platform. LV, left ventricle; RV, right ventricle.

The cardiac phantom (IBA QUASAR, London, Ontario, Canada) presented in this work was designed to mimic the human ventricles of the heart while combining MRI‐compatibility constraints and being compatible with the QUASAR^TM^ MRI^4D^ Motion Phantom (IBA QUASAR, London, Ontario, Canada) to facilitate its use. The five main elements composing this phantom are as follows: (1) a deformable heart model, (2) a rigid 3D printed section filled with (3) a 4 ppm Mn^2+^ contrast solution, (4) a moving piston, and (5) four integrated dosimeters (Figure 1a,b). This cardiac phantom is used by connecting its piston (4) to the motor of the motion phantom, and as a consequence, they are always connected together during experimental work (Figure 1c). A detailed description of the elements (1 – 4) can be found in the Supporting Material.

The dosimeters (5) in this phantom are Plastic Scintillation Detectors (PSDs): Hyperscint RP system using a customised FLEX Scintillation Detector four channels connected to a RP‐200 reader (Medscint, Quebec, Quebec, Canada). These PSDs were manually integrated (and glued) in the silicon of the heart model (1). They are based on scintillation technology, are water‐equivalent, and allow to perform real‐time dosimetry in addition to dose accumulation.^23^ Each of them contains a scintillator (cylindrical sensitive volume: *D* = 1 mm; length = 1 mm) and an optical fiber, which are both wrapped in a plastic envelop (*D* = 2 mm). After appropriate calibration,^24^ each PSD enables the measurement of the dose deposited on their scintillator during a predefined exposure time (referred to as measurement frequency). The dose deposition information is transferred from the scintillator to the HYPERSCINT^TM^ RP reader for measurement via the optical fiber.

In this work, the cardiac phantom was filled with 490 mL of contrast solution, corresponding to a relaxed state obtained for motor position 0 mm. The motion and the position of its piston were preprogrammed using the QUASAR software (QUASAR Respiratory Motion^TM^, version: 4.2.13) and ranging from the motor positions −15 mm to +5 mm to allow contraction and expansion of the heart model. When dosimetric testing was performed, the phantom was placed on top of a motion platform — QUASAR^TM^ Motion MR Platform (IBA QUASAR, London, Ontario, Canada) — to facilitate the application of a respiratory motion on it (Figure 1c). Unless stated otherwise, experiments were performed on a 1.5 T Unity MR‐linac (Elekta AB, Stockholm, Sweden).

### 2.2 Phantom characteristics

#### 2.2.1 MRI properties — T1 and T2 values

To evaluate the MRI properties of the phantom, we measured its T1 values using a gold standard single‐slice single‐echo inversion recovery sequence (TR = 10000 ms, TE = 20 ms, and TI = [50, 200, 500, 900, 1400, 1900, 2500, 3000] ms) and its T2 values using a gold standard single‐slice single‐echo spin echo sequence (TR = 10000 ms and TE = [10, 60, 90, 130, 160, 250, 300, 360, 460, 510, 760] ms). We used the same transverse slice with an (acquired) in‐plane resolution of 1.2 × 1.2 mm^2^ and a slice thickness of 3 mm. Data were acquired at room temperature (average: 21.9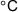) on a 1.5 T Ingenia MRI system (Philips Healthcare, Best, the Netherlands). We then computed the T1 and T2 maps using an in‐house script (code available online: https://github.com/oscarvanderheide/goldstandard_T1_T2). We finally manually segmented three regions of interest (ROIs) in these maps: the left ventricle's cavity filled with contrast solution (ROI 1), the left ventricle's wall (ROI 2), and the right ventricle's wall (ROI 3). The T1 and T2 values of each ROI were defined as their averaged values and compared to the relaxation times of the blood and of the myocardium in a human heart.

#### 2.2.2 2D deformation and mechanical behavior

To evaluate the 2D deformation and mechanical behavior of the phantom, we performed an MRI‐based analysis inspired by a dynamic mechanical analysis (DMA). A DMA is performed by applying a sinusoidal stress on a material and by measuring its resulting strain. In our case, we applied a sinusoidal waveform on the piston of the cardiac phantom and measured the resulting deformation of its heart model. This analysis allowed to evaluate (1) a potential phase lag of deformation with respect to the motion applied on the piston and (2) a potential hysteresis behavior of the heart model, which are two important indicators of visco‐elastic behavior. To perform this analysis, we proceeded with the following steps.

**FIGURE 2:**
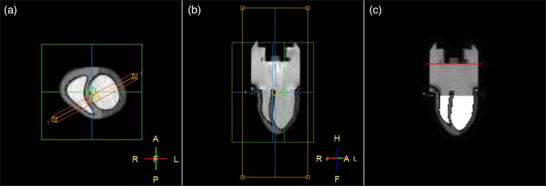
Selected slice for data acquisition and analysis visual representation. (a) Transverse view with the selected slice to acquire pictured. (b) Example of a resulting frame of the 2D bFFE cine scans. (c) Analysis visual representation. The red line shows the determined piston position and the white areas correspond to the segmented ventricular cavities.

We first acquired 2D bFFE cine scans (acquisition rate: 10.5 Hz, FOV: 280 × 108 mm^2^, TR/TE: 3.7/1.84 ms, resolution: 1.6 × 1.6 × 9 mm^3^, CS factor: 2, partial Fourier factor: 0.75, flip angle: 50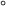) while applying a sinusoidal waveform on the piston (waveform 1: *f* = 60 bpm, *A* = 10 mm; waveform 2: *f* = 60 bpm, *A* = 7 mm; waveform 3: *f* = 80 bpm, *A* = 7mm). To picture the maximum deformation of the two ventricles simultaneously, we acquired a coronal slice with angle = −28 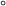 in the foot‐head direction (Figure 2a,b). Considering a deformation state of the heart model linked to a given motor position, that is, a given piston position, we also acquired 2D bFFE cine scans every millimeter from static motor position −15 to +5 mm, and from +5 to −15 mm to simulate a phase information (piston pushed in or pulled out, i.e., diastole or systole). 250 frames were acquired when motion was applied, while 100 frames were acquired under static conditions. All the scans were acquired before PSDs integration. We then determined, for each frame, the piston position and we quantified the heart model deformation (Figure 2c). The piston position was determined by locating the change in intensity at the interface piston – contrast solution in the image. The heart model deformation was quantified by segmenting the ventricular cavities using the marching squares method and by calculating the resulting areas. To evaluate the potential phase lag of the heart model deformation with respect to the piston motion, we used the dynamic cine scans acquired to compare them over time. To evaluate the potential hysteresis behavior of the heart model, we plotted a dispersion graph (in analogy to a stress–strain curve; a piston position–area ventricles curve) and we took into account the phase information of our data. For the dynamic cases, each piston position was periodically reached according to the waveform applied. As a consequence, to determine the area of the ventricles for a given piston position, we computed the (mean, std) area of the ventricles over the associated frames. We used the frames acquired once steady state of the magnetization was reached and after stabilization of the motion applied (one period): the first frame considered was between the 50^th^ and 60^th^ ones for each cine scan. Additionally, the result obtained was only used if the piston position was determined at least five times for all the frames considered. For the static case, we also computed the (mean, std) area for each given position using the last 50 dynamic frames to match the approach used in the dynamic cases. We then fitted a linear regression model to evaluate the elastic behavior of the heart.

#### 2.2.3 3D deformation, mechanical behavior, and stroke volume

**FIGURE 3:**
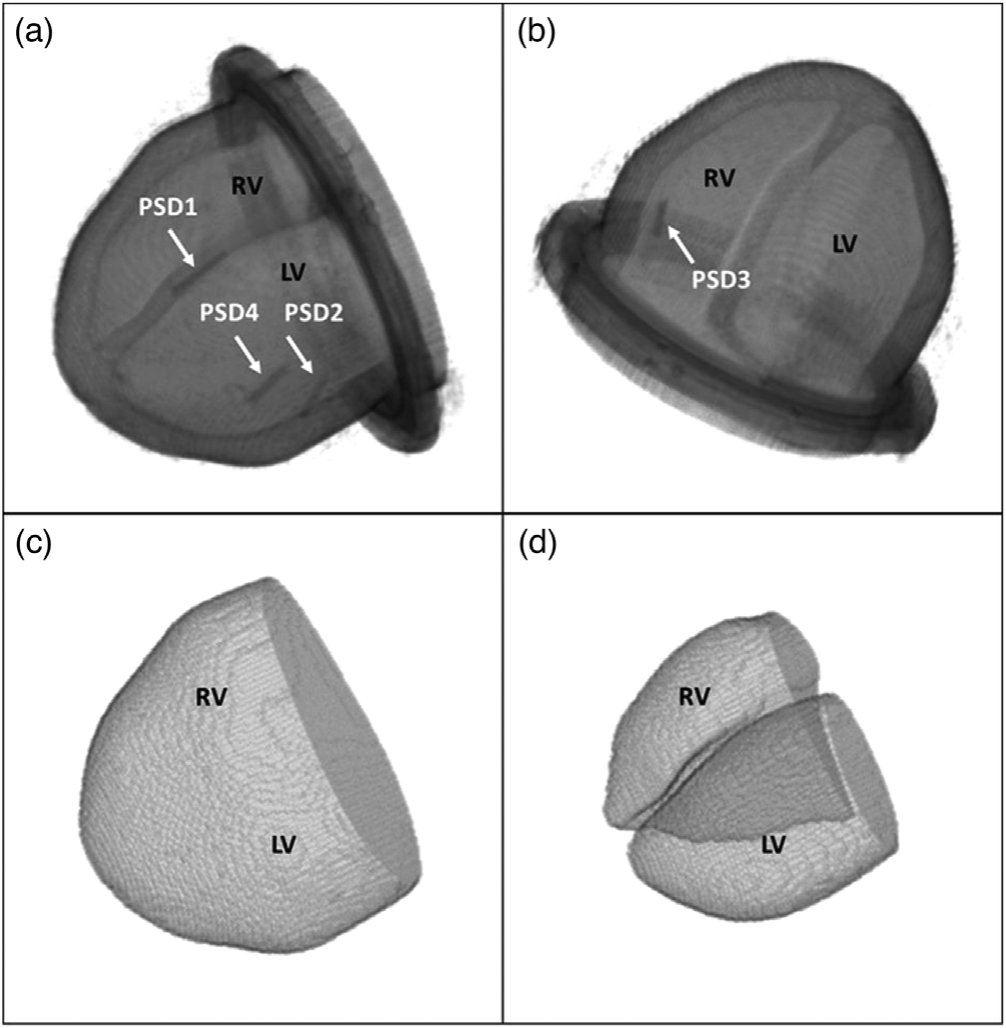
3D rendering of a 3D MRI scan acquired after PSDs integration (motor position = −5 mm) and its segmented VOIs. (a, b) 3D scan — the four PSDs are visible. (c) VOI 1: heart model filled with contrast solution. (d) VOI 2: cavities only filled with contrast solution. LV, left ventricle; RV, right ventricle; PSD, plastic scintillation detector.

To evaluate the 3D deformation and mechanical behavior of the phantom, and to compute its stroke volume, we first acquired a 3D scan each millimeter from the motor positions −15 to +5 mm to obtain 3D volumes of these corresponding deformation states of the heart model (Figure 3a,b). We used a turbo spin echo (TSE) sequence (TR: 1000 ms, ETL: 30, effective TE: 76 ms, resolution: 1 × 1 × 1 mm^3^, SENSE factor: 2, partial Fourier factor: 0.7, NSA: 2). To evaluate the impact of the integrated PSDs on the deformation, data were acquired before and after their integration. The integration of the PSDs in the heart model, however, required to disassemble and re‐assemble the phantom, implying to refill it with contrast solution as similarly as before integration. For each 3D scan acquired, we then segmented two volumes of interest (VOIs) (Figure 3c,d): the heart model with its cavities filled with contrast solution (VOI 1) and a sub‐segmentation of the cavities only (VOI 2). We then plotted a dispersion graph in analogy to a stress–strain curve (a motor position–VOI curve) for each segmented VOI before and after PSDs integration. We then fitted a linear regression model to evaluate the elastic behavior of the heart model and we computed the stroke volume for the combined ventricles of the cardiac phantom. We lastly performed a deformable image registration (DIR)^25^ to determine the deformation of the cardiac phantom at each point. Knowledge on material properties was used to constraint the algorithm (Section 3 and Supporting Material). We validated the registration by computing the target registration error (TRE) on the PSD centroids before and after registration. PSD centroid positions were defined in the 3D scans after PSDs integration and positions defined were used for validation on the 3D scans before PSDs integration.

### 2.3 Phantom dosimetric capabilities

#### 2.3.1 Treatment planning: STAR plan

To create the STAR plan, we first acquired a CT scan of the cardiac phantom (motion phantom: motor position = −5 mm, motion platform: motor position = 0 mm) with a Brilliance CT Big Bore (Philips Healthcare, Best, the Netherlands). We then used Elekta's Monaco treatment planning system (TPS) (version: 6.2.1.0) to define our main structures of interest: GTV, PTV, ventricular cavities, heart model, PSD scintillators, and motion platform. We defined the GTV to follow the myocardial wall of the left ventricle and to include PSD 1 and PSD 4. We assigned a PTV with 2‐mm isotropic margins around the GTV to have PSD 2 localized at its edge. We finally created our STAR plan: a step and shoot IMRT plan (15 beams) with a prescribed dose of 1 × 25 Gy (V95% prescribed dose ≥ 99%) that we scaled down with a factor 2 to reduce the delivery time. Before delivery, we adapted our plan based on the daily MRI scan of the cardiac phantom (motion phantom: motor position = −5 mm, motion platform: motor position = 0 mm) to obtain a reference static planned dose, and we defined a gating structure used for gated delivery. This gating structure was defined as a rectangular prism and was placed at the interface contrast solution–silicon on top of the left ventricle to depict the motion experienced by the GTV/PTV.

#### 2.3.2 Reliability of PSD measurements

To evaluate the reliability of the PSD measurements, we first calibrated the PSDs by performing a spectral calibration and a dose calibration. Spectral calibration was performed following a similar approach as presented in a previous work.^24^ Dose calibration was performed by creating a step and shoot IMRT plan (15 beams) aiming to deliver a dose as homogeneous as possible on the PSDs. To create robustness to setup uncertainty, we constrained the plan by creating 2‐mm isotropic margins around the PSD structures and by forcing homogeneity into these new structures. We then tested the repeatability, the linearity, and the dose rate dependency of the dose measured by the PSDs. This testing was performed using a measurement frequency for the PSDs of 14.7 Hz, which was the highest frequency available in Medscint's software (HYPERDOSE RP, version: 1.5.2), and using a 10 × 10 ${\mathrm{cm}}^{2}$ beam at gantry 0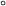. To test the dose repeatability, each measurement was performed five times. To test the dose linearity, we evaluated the dose measured by the PSDs for a delivery of [100, 200, 500, 800, 1000] MU. To test the dose rate dependency, we delivered 50 MU at dose rates from 50 to 400 MU/min with an increment of 50 MU/min.

#### 2.3.3 Dosimetric impact of motion and motion mitigation

To evaluate the dosimetric impact of motion and motion mitigation (using gating) on the dose measured by the PSDs, we delivered the STAR plan created considering different cases. These cases were as follows: (1) a delivery with no motion applied on the cardiac phantom (referred to as static), (2) a delivery with motion applied but not mitigated (referred to as motion), and (3) a delivery with motion applied and mitigated using gating (referred to as gating). We compared this different cases when the motion applied was cardiorespiratory motion (referred to as subcase CRM) or cardiac motion (referred to as subcase CM).

The setup used here allowed to apply on the cardiac phantom the cardiac motion (waveform = sin, *f* = 60 bpm, *A* = 10 mm) through the motion phantom and the respiratory motion (waveform = $\cos^{4}$, *f* = 12 bpm, *A* = 20 mm) through the motion platform. When cardiorespiratory motion was applied, the two waveforms were synchronized thanks to the dual phantom control feature in the QUASAR software. When only cardiac motion was applied, the motion platform was static (position = 0 mm). When no motion was applied, both the motion phantom and motion platform were static (respectively at position = −5 mm and position = 0 mm). For the gated deliveries, we used the Elekta Unity's Comprehensive Motion Management (CMM) clinical workflow.^18^ The gating was performed by acquiring two orthogonal interleaved 2D scans (acquisition rate ∼6 Hz in SI direction) centered on the predefined gating structure, and by checking if the position of this gating structure was within our tolerance. We chose to check its position against given displacement thresholds in the SI direction: the beam was disabled when the estimated position of the gating structure was not within our tolerance or could not be extracted with sufficient confidence. A nonisotropic gating threshold was defined, in which the irradiation was paused at a displacement >3.8 mm in inferior direction for CRM, and >0.5 mm in superior direction for CM with respect to the reference position. These thresholds were aimed at a duty cycle of ∼50%. To use the CMM workflow for the gated deliveries, the original STAR plan needed to be adapted twice (subcases CRM and CM): setup and beams delivered were identical, but planned doses obtained were different as the PSD structures were recontoured for each adapted plan. As a consequence, for each subcase, the gated delivery was performed first and the associated adapted plan was re‐delivered for the static and motion cases.

We then analyzed the results of our deliveries by comparing the cumulative doses and the dose rates measured by the PSDs for the different cases. We compared the cumulative doses in the static cases to the TPS. Concurrently, we evaluated the impact of the PSD structures position in the TPS on their planned doses by calculating the percentage difference between the maximum and minimum planned doses in each of them. We then compared the cumulative doses received in the motion and gating cases to the static cases. We finally compared the dose rates to the motion applied over time in the motion and gating cases.

## 3 RESULTS

### 3.1 Phantom characteristics

#### 3.1.1 MRI properties — T1 and T2 values

**FIGURE 4:**
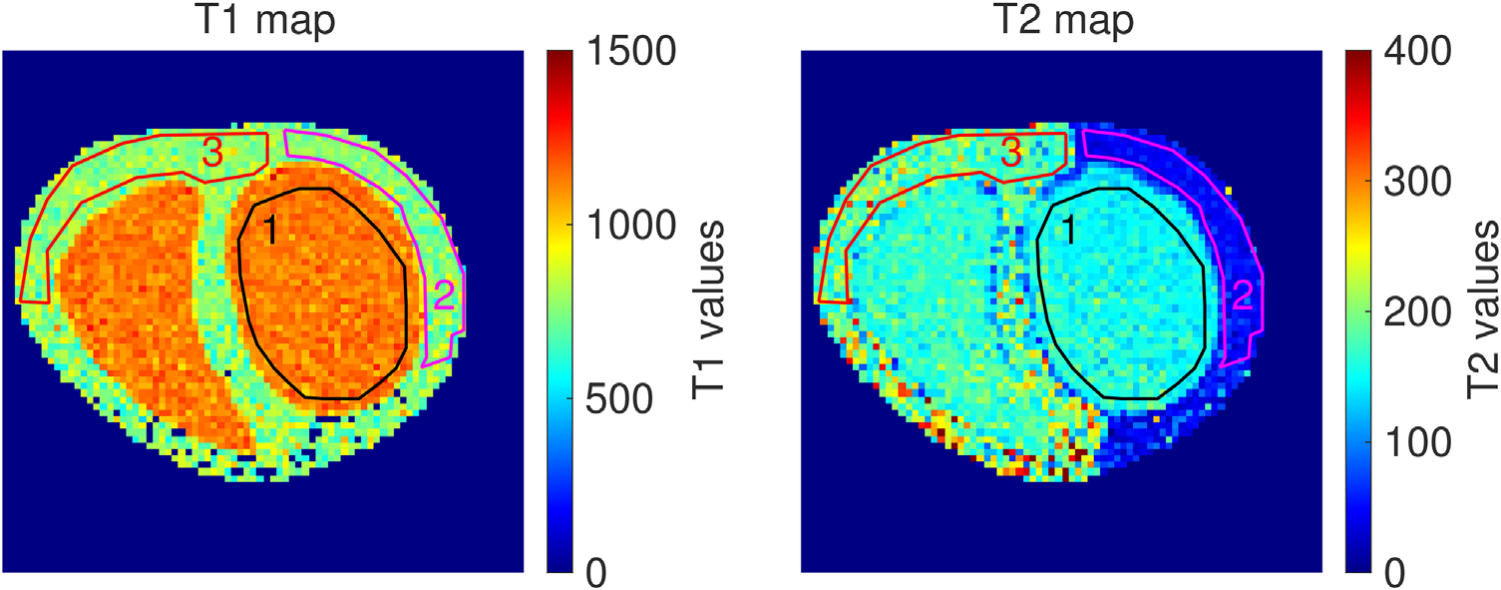
T1 and T2 maps with the drawn ROIs. Left: T1 map, right: T2 map, black: ROI 1, magenta: ROI 2, red: ROI 3.

Table 1 shows the mean T1 values and the mean T2 values obtained for the contrast solution (ROI 1), the left ventricle's wall (ROI 2), and the right ventricle's wall (ROI 3). Literature values^26^, ^27^, ^28^, ^29^, ^30^, ^31^, ^32^ for corresponding blood and myocardium in the human heart are also given. Figure 4 shows the T1 and T2 maps used to compute the mean T1 and T2 values of each ROI.

**TABLE 1:** T1 and T2 values of the cardiac phantom and in vivo values.

	T1 values phantom	T1 values	T2 values phantom	T2 values
	(mean ± std ms)	in vivo (ms)	(mean ± std ms)	in vivo (ms)
ROI 1	1130 ± 44	[1200–1500]^26^, ^27^	155 ± 16	[100–200]^26^
ROI 2	763 ± 76	∼1000^27^, ^28^, ^29^	49 ± 12	[50–55]^30^, ^31^, ^32^
ROI 3	775 ± 69	∼1000^27^, ^28^, ^29^	185 ± 35	[50–55]^30^, ^31^, ^32^

*Note*: ROI 1: contrast solution (blood); ROI 2: left ventricle's wall (myocardium); ROI 3: right ventricle's wall (myocardium).

#### 3.1.2 2D deformation and mechanical behavior

**FIGURE 5:**
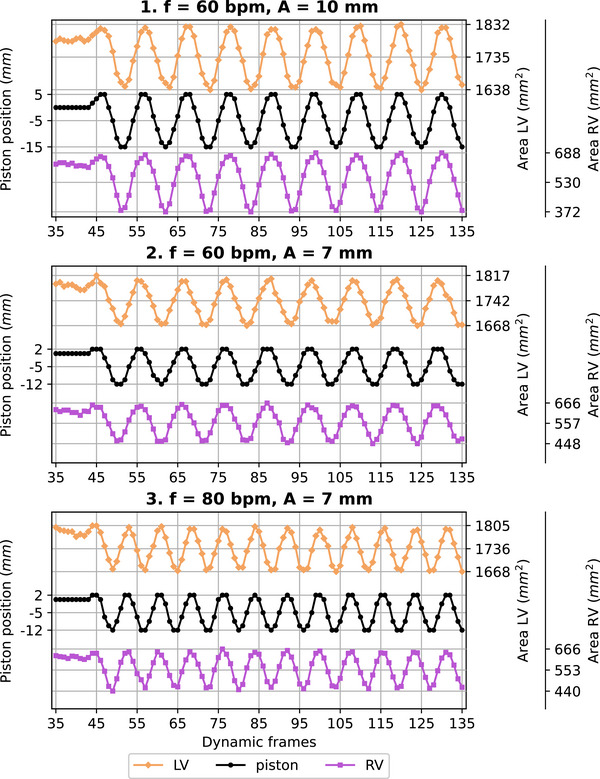
Piston positions and ventricular areas are plotted for each dynamic frame of the cine scans acquired while motion was applied for three different waveforms. Motion was started between the 40^th^ and 50^th^ dynamic frames once the steady state of the magnetization was reached. LV, left ventricle; RV, right ventricle.

**FIGURE 6:**
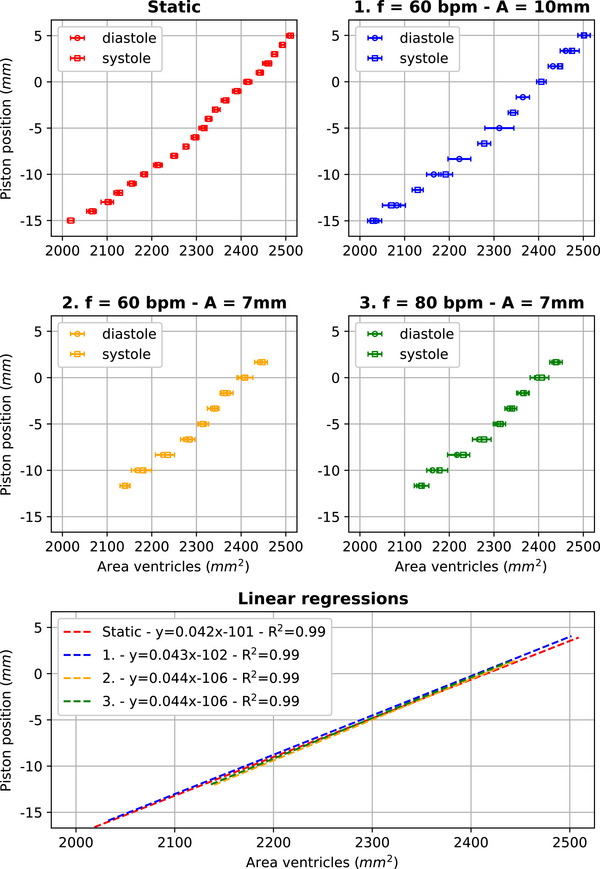
Piston position–area ventricles curves for the different motion patterns tested and their linear regression. Error bars represent the standard deviations.

Figure 5 shows the results obtained when piston position and areas (left and right ventricular cavities) of the heart model are plotted over time in the dynamic cases (motion applied during data acquisition). The type of response observed over time for the left and right ventricles when the piston was moving is similar. Figure 6 shows the correlation between piston positions and ventricular areas. With the similar type of response observed in Figure 5 for the two ventricles, we here plotted a combined area for the two of them. For the static case, no difference in ventricular area is observed when a piston position is reached in diastole, that is, piston pushed in, or in systole, that is, piston pulled out. For the three dynamic cases, a minor difference in ventricular area is observed when a piston position is reached in diastole or in systole. In all cases, a positive linear relationship is obtained between piston position and ventricular area (*R* ^2^ = 0.99) with comparable slopes (∼0.04 mm^−1^).

#### 3.1.3 3D deformation, mechanical behavior, and stroke volume

**FIGURE 7:**
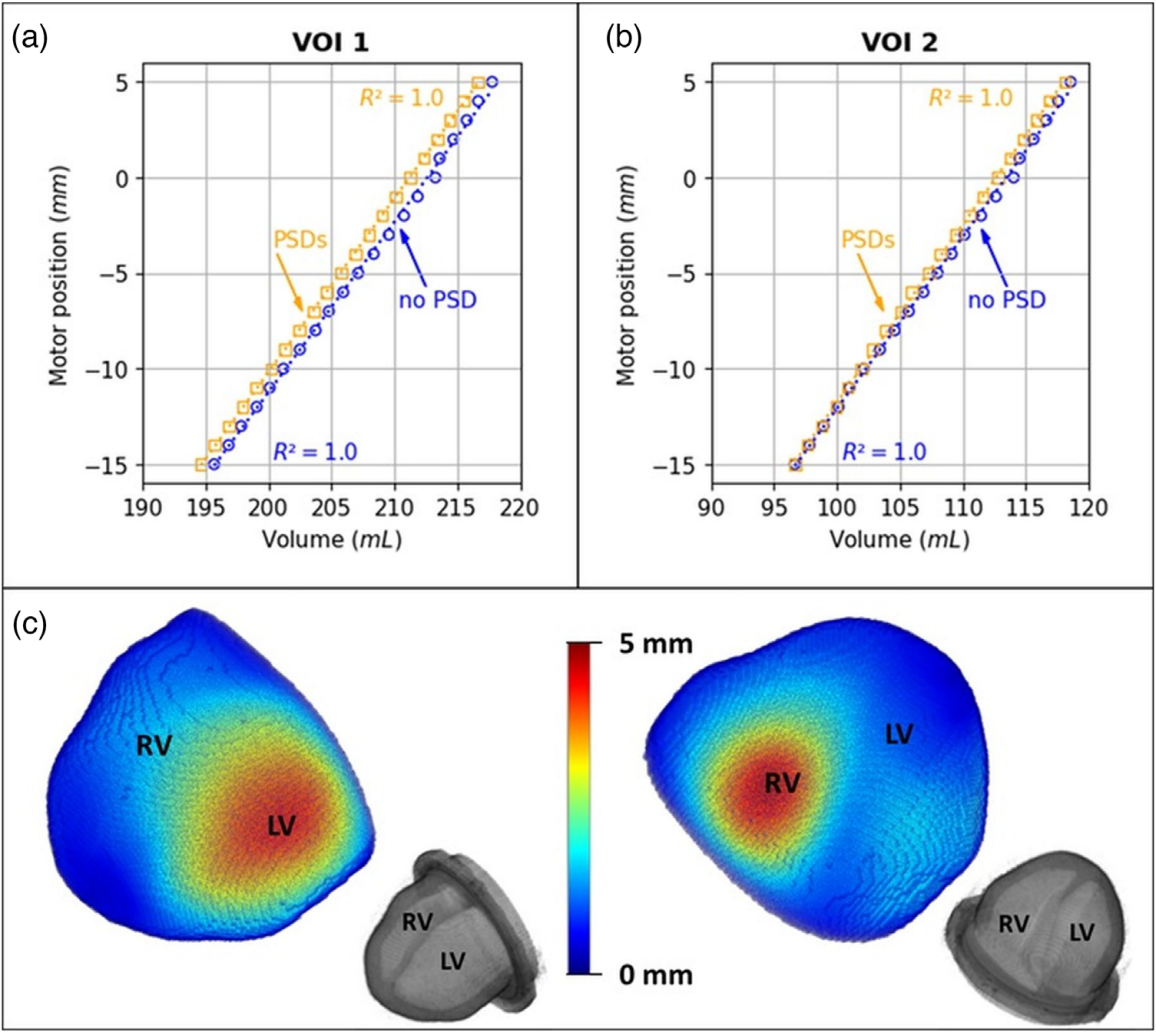
3D deformation of the cardiac phantom. (a, b) Motor position‐VOI (VOI 1 or VOI 2) curves before and after PSDs integration with their respective linear regressions. (c) 3D rendering of the magnitudes computed from the smallest (motor position = −15 mm) to the largest (motor position = +5 mm) deformation states based on the deformation vector fields obtained (DIR). 3D rendering of an MRI scan (motor position = −5 mm; before PSDs integration) is also shown for orientation. LV, left ventricle; RV, right ventricle.

Figure 7a,b shows the correlation between the motor positions and the volumes of each VOI (VOI 1: heart model with cavities filled with contrast solution; VOI 2: cavities only). The motor positions and the volumes of each VOI are linearly correlated whether the PSDs were integrated or not ($R^{2}=1.0$). The volumes of VOI 1 and VOI 2 obtained for each motor position are higher before PSDs integration than after. However, the largest absolute percentage difference computed for VOI 1 is 0.94% and for VOI 2 1.1%. The stroke volume obtained before PSDs integration is 21.9 mL against 21.4 mL after PSDs integration. Figure 7c shows the magnitudes computed from the smallest (motor position = −15 mm) to the largest (motor position = +5 mm) deformation states based on the deformation vector fields obtained after DIR. Two hot spots of deformation were observed: one in the left ventricle and one in the right ventricle. The maximum deformation observed (largest magnitude) was of 5 mm. The deformation observed at the apex of the heart model did not exceed a magnitude of 0.9 mm. TRE measured on PSD centroids before and after registration were as follows: PSD1: TRE_before_ = 1.6 mm, TRE_after_ = 1.9 mm; PSD2: TRE_before_ = 4.2 mm, TRE_after_ = 0.6 mm; PSD3: TRE_before_ = 3.1 mm, TRE_after_ = 1.3 mm; PSD4: TRE_before_ = 4.2 mm, TRE_after_ = 1 mm.

### 3.2 Phantom dosimetric capabilities

#### 3.2.1 Reliability of PSD measurements

The measured dose per prescribed MU and per dose rate was very similar for each case with a coefficient of variation CV ≤ 0.31%. The measured dose as a function of prescribed MU gave excellent linearity (*R* 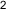 = 1.0). The dose rate dependency as the coefficient of variation over the averaged 5 repeated measurements by dose rate was ≤0.72%.

#### 3.2.2 Dosimetric impact of motion and motion mitigation

**FIGURE 8:**
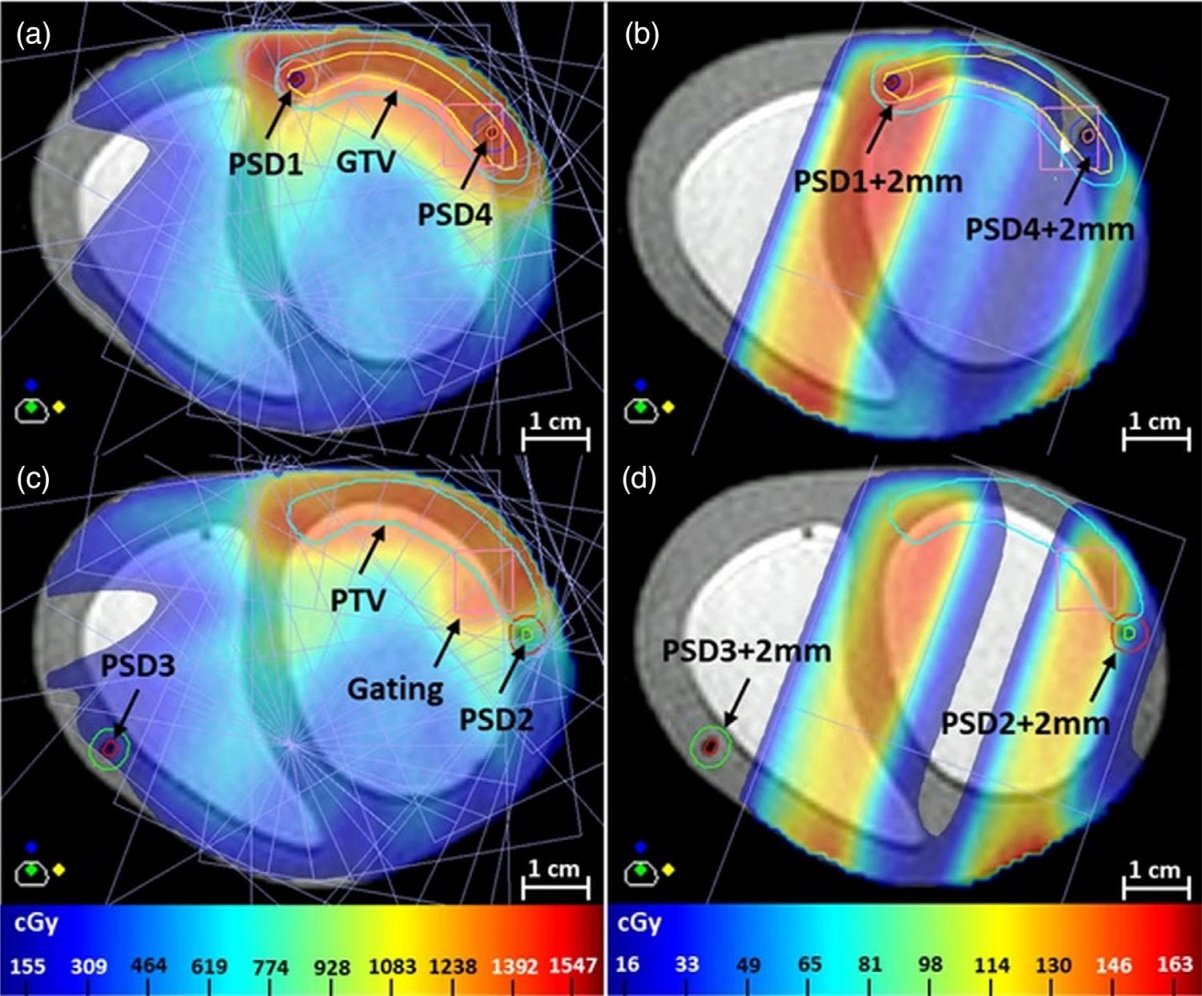
Planned dose distribution given by the TPS for the entire adapted STAR plan (a, c) and for one of its beam (gantry angle = 20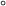) (b, d).

**FIGURE 9:**
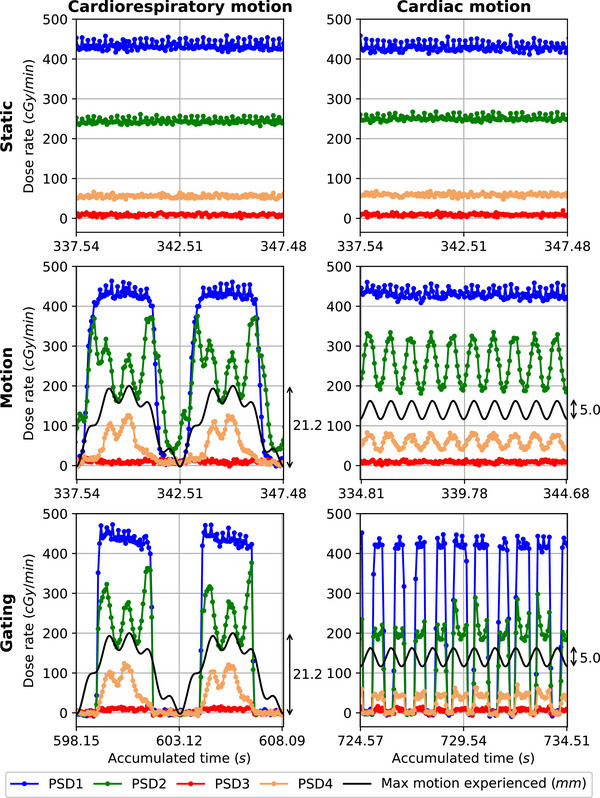
Example of the dose rates measured by the PSDs during the delivery of one beam (gantry angle = 20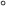) of the STAR plan — the maximum motion experienced is the maximum motion experienced by the heart model.

Figure 8 shows the planned dose distribution given by the TPS for the entire adapted STAR plan and for one of its beam (gantry angle = 20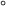). Figure 9 gives an insight on the dose delivered in real‐time to the PSDs during the delivery of one beam of the STAR plan. For this beam, PSD3 was outside the direct field (∼ 8 mm from field edge) while PSD1, PSD2, and PSD4 were in‐field (cf. Figure 8b,d). PSD1 was in a high‐dose region, PSD2 in a gradient region, and PSD4 in a low‐dose region. For the static cases, a constant dose rate was measured by the PSDs over time. For the motion and gating cases, they measured a fluctuating dose rate in phase with the motion pattern. While they all experienced the respiratory motion in subcase CRM, the cardiac motion experienced by each of them was dependent on their location on the heart model, that is, on the heart model's 3D deformation in response to the cardiac motion applied. Additionally, for the gating cases, all PSDs measured a dose rate of 0 cGy/min when the beam was turned off during gating.

Total delivery time for static and motion cases was about 9 minutes while they were about 16 minutes for CRM‐gating case and 21 minutes for CM‐gating case, giving a duty cycle of 56% and 43% for CRM‐gating and CM‐gating cases, respectively. Table 2 shows for the different cases the cumulative doses obtained and percentage errors calculated. The table also shows the percentage differences calculated between the maximum and the minimum planned dose in each PSD structure in the TPS.

**TABLE 2:** Cumulative doses and percentage differences or errors for the STAR plan delivered.

Cardiorespiratory motion (CRM)								
	Cumulative doses (cGy)				Percentage differences/errors (%)			
	PSD1	PSD2	PSD3	PSD4	PSD1	PSD2	PSD3	PSD4
TPS	1408	865	192	1388	*3.1*	*14.2*	*20.7*	*2.5*
1. Static	1381	909	210	1356	−1.9^a^	5.1^a^	9.4^a^	−2.3^a^
2. Motion	986	1014	208	974	−28.6	11.5	−0.8	−28.2
3. Gating	1375	884	203	1350	−0.4	−2.7	−3.1	−0.5

Cardiac motion (CM)								
	Cumulative doses (cGy)				Percentage differences/errors (%)			
	PSD1	PSD2	PSD3	PSD4	PSD1	PSD2	PSD3	PSD4
TPS	1408	881	189	1385	*2.8*	*11.3*	*11.5*	*1.7*
1. Static	1374	914	205	1350	−2.4^a^	3.8^a^	8.5^a^	−2.5^a^
2. Motion	1367	912	206	1340	−0.6	−0.2	0.4	−0.7
3. Gating	1382	921	211	1340	0.6	0.8	3.1	−0.7

*Note*: In the column “Percentage differences/errors (%)”, italic values are the percentage differences calculated between the maximum and the minimum planned dose in the TPS, while the other values are percentage errors of the cumulative doses.

^a^ Static cases were compared to TPS, while motion cases and gating cases were compared to static cases.

## 4 DISCUSSION

In this work, we present a cardiac phantom supporting end‐to‐end testing for heart radiotherapy on a 1.5 T MR‐linac.

The MRI properties of the phantom were compared to the in vivo properties of the heart and resulted, on average, in an agreement of T1 of −16.3%, −23.7%, and −22.5%, and of T2 of 3.3%, −6.6%, and 252.4% for the blood and the contrast solution, the myocardium and the left ventricle's wall, and the myocardium and the right ventricle's wall, respectively. The relatively high standard deviations associated (Table 1) could be explained by the low signal‐to‐noise ratio caused by the small voxel size, which was used to prevent partial volume effects for the cardiac wall structure. The significance of the differences between phantom and in vivo MRI properties would be dependent on the specific cardiac application. Adjustments in the material composition could be made to better match physiological properties if needed. Although a lengthening of the T1 value for the three heart model's elements and a shortening of the T2 value for the right ventricle would be preferred for cardiac MRI sequence optimization, the current MRI properties of the phantom were satisfactory for the study.

The 2D deformation and mechanical behavior of the phantom were then assessed in a dynamic and static settings. Compromises on image quality needed to be made to reach a sufficient acquisition rate (10.5 Hz), which led to a limited image resolution and visible (banding and chemical shift) artifacts (Figure 2). Nonetheless, the implemented segmentation approach remained robust with, in the static case, a maximum coefficient of variation of 0.66%. For the three waveforms applied on the piston, the ventricular area of each cavity (area LV and area RV) oscillated with the same periodicity and in phase with the piston motion (Figure 5). The “piston position–area ventricles curve” showed only minor differences (remaining in the range of the standard deviations) in ventricular areas between diastole and systole for a given piston position (Figure 6). Given that no phase lag of the heart model deformation with respect to the piston motion (1) and no hysteresis behavior (2) were observed: the heart model did not exhibit viscous behavior. Additionally, linear relationships (*R* ^2^ = 0.99) with similar slopes (∼0.04 mm^−1^) were obtained in all cases: the heart model exhibited a symmetric elastic behavior, which was independent of the motion pattern that we tested. This mechanical behavior of the heart model is nevertheless different from the one of the human heart. In a previous study,^33^ the passive human left ventricle myocardium has been characterized as a nonlinear, anisotropic, and viscoelastic material. Although a more realistic mechanical behavior of the heart model could be of interest for specific cardiac applications, its symmetric elastic behavior makes the phantom's motion easy to model and, therefore, facilitates end‐to‐end testing.

The 3D elastic behavior of the heart model was confirmed by the linear relationships obtained between motor positions and VOIs (VOI 1: heart model with cavities filled with contrast solution, and VOI 2: sub‐segmentation cavities only) before and after PSDs integration (*R* ^2^ = 1.0). Differences in volume observed before and after PSDs integration for each VOI were considered negligible. This negligible difference in volume for VOI 2 showed the reproducibility of the cardiac phantom filling (phantom emptied and refilled to integrate the PSDs) and for VOI 1, the limited impact of the integrated PSDs on the heart model's 3D deformation. Difference in stroke volume was also consistent with the other differences observed. Additionally, the DIR algorithm used to quantify the 3D local deformation showed a maximum magnitude of deformation between the two extreme deformation states of 5 mm. TREs computed to validate the obtained deformation vector fields were of the order of 1 mm after registration. The accurate estimation of these reported TREs was limited by the scan resolution (1 × 1 × 1 mm^3^) and by the uncertainty on the PSDs centroid position. The PSDs centroid position is intrinsically uncertain as segmenting the PSDs accurately was challenging due to partial volume effect. These reported TREs also rested on the assumption that the negligible impact of the integrated PSDs on the global heart model's deformation was also valid locally. For those reasons, we considered the resulting accuracy of the registration acceptable.

The dosimetric capabilities of PSDs on 1.5 T MR‐linacs have been demonstrated before in simpler setups, and our measurements (repeatability: CV ≤ 0.31%, linearity: *R* ^2^ = 1.0, dose rate dependency: CV ≤ 0.72%) give values of the same order of magnitude than the ones found in the literature (repeatability: CV < 0.5%,^24^, ^34^, ^35^ linearity: *R* ^2^ = 1.0,^24^, ^34^ dose rate dependency: <0.3%^24^). Their high reliability in our complex setup was shown, facilitating the assessment of the dosimetric impact of motion and motion mitigation. The percentage differences calculated between the maximum and minimum planned doses in each PSD structure on the TPS were rather high (between 1.7% and 20.7%) and resulted in rather high percentage errors (between −2.5% and 9.4%) computed on the cumulative doses for the static cases with respect to the TPS. These results can be due to the dose calculation uncertainty of the TPS (Monte‐Carlo‐based, taking into account magnetic field effects^36^), and to the contouring of the PSD scintillators (partial volume effect, exact position in the PSD envelopes) on the TPS. For the subcase CRM, the dosimetric impact of motion and motion mitigation on the dose deposited on the PSDs is clearly noticeable when computing percentage errors on the cumulative doses for the motion and gating cases with respect to their static case. In the motion case, PSD1 and PSD4 were periodically out‐field (−28.6% and −28.2% error, respectively) while PSD2 was periodically in‐field (11.5% error), and PSD3 remained in a low‐dose region (−0.8%). In the gating case, motion was mitigated and percentage errors dropped for PSD1, PSD2 and PSD4 (−0.4%, −2.7%, and −0.5% error, respectively). For the subcase CM, percentage errors computed on the cumulative doses for the motion case with respect to its static case showed similar measured doses, and showed that the dosimetric impact of the more subtle cardiac motion on the total delivered dose is limited. The motion mitigation is, as a consequence, not improving the dosimetric outcome of the delivery.

Nonetheless, the choices made to perform this dosimetric testing also have their limitations. The manual integration of the PSDs within the cardiac phantom was user‐dependent and could lead to the presence of air pockets, which can change the dose measured by the PSDs.^24^ The delivery of a complete homogeneous dose to the PSD scintillators during dose calibration was also challenging: the constraints applied to optimize the IMRT plan were difficult to respect, and the position of the PSD scintillators inside the heart model was uncertain. A more standardized approach to integrate the PSDs and to localize the imaged PSD scintillators could be desired to improve dosimetric outcomes. Additionally, dosimetric testing was performed with the application of a simulated cardiac motion: a sinusoidal waveform at a frequency of 60 bpm and an amplitude of 10 mm. However, the in vivo cardiac cycle is composed of a diastolic phase that is significantly longer than the systolic phase, and the resulting cardiac motion can be observed at higher frequencies than 60 bpm.^14^ The use of this simplistic cardiac motion allowed to have a straightforward estimation of the heart model deformation during delivery (Subsection 3.1). The application of more realistic waveforms (shape and frequency) could, nonetheless, improve the realism of the testing. On the same note, although the maximum magnitude of deformation observed in the phantom (5 mm) matches the average motion of the target volume induced by cardiac contraction in VT patients,^15^ some studies reported larger cardiac‐induced motion amplitude with, for example, a 3D cardiac motion of the left ventricle on average of 10.2 ± 2.7 mm for VT patients,^15^ or even larger for other cardiac substructures in healthy volunteers.^14^ To better depict the range of cardiac‐induced motion magnitude of the human heart, the heart model's deformation magnitude could be further enlarged. This enlargement would also allow to test the dosimetric impact of cardiac motion for larger magnitude of motion. Although the dosimetric impact of cardiac motion was limited in this work, simulations^37^ showed deviation of PTV $D_{95}$% up to −3.8% due to cardiac motion with motion magnitudes between 7.5 and 11.3 mm. Transferability of our results to a real human heart could be consequently further investigated. Another step to improve the realism of our testing for future work would be to adjust the surrounding environment of the heart model. This new setup could integrate the heart model into a body scaffold that mimics the lung environment and allows the placement of an ICD. As a consequence, imaging (banding artifacts) and dosimetric (electron return effect) conditions would better mimic the patient condition and allow further end‐to‐end testing.

Although having its own limitations, the features offered by this novel cardiac phantom are noteworthy. To the best of our knowledge, only another cardiac phantom^21^ combines MRI compatibility with dosimetric possibilities, which are necessary features for end‐to‐end testing on MR‐linacs. These two phantoms use scintillation technology to perform real‐time dose measurements and dose accumulation, and they are both able to show the dosimetric impact of motion and motion mitigation in real‐time. They, however, diverge in design. While the other phantom consists of two interchangeable modules (containing the cardiac substructures) that are rigidly displaced in one direction, our cardiac phantom is composed of a heart model that deforms in three directions. The deformability of this novel cardiac phantom allows to mimic the contracting behavior of a human heart and, consequently, enables end‐to‐end testing for heart radiotherapy with realistic deformable cardiac motion.

## 5 CONCLUSION

In conclusion, we showed that this deformable and MRI‐compatible cardiac phantom has good MRI contrast, and that its heart model exhibits elastic behavior. Its integrated PSDs offer real‐time and multi‐point dose measurements, and we demonstrated their reliability. We successfully showed that this cardiac phantom opens new possibilities to support the development and validation of new cardiac‐optimized workflows on MR‐linacs.

## CONFLICT OF INTEREST STATEMENT

Co‐authors K. I. Penev and N. Hartman are a former and a current employee of IBA QUASAR, respectively. Co‐author Y. LeChasseur is an employee of Medscint.

## Supporting information



Supporting Information
