# Supplementary material for: Characterization of a deformable beating cardiac phantom with real‐time dosimetric capabilities for validation of MRI‐guided heart radiotherapy
Source: Med Phys. 2026 Feb 12;53(2):e70313. doi: 10.1002/mp.70313 (PMC12900995; doi:10.1002/mp.70313)
Supplement: Supplementary file 1 — Supporting Information [file MP-53-0-s001.pdf]

Supporting Material - Phantom Design

The heart model *(1)* was derived by imaging acquisition of a human heart to mimic the structure of the human ventricles. It represents the myocardium of the heart, and two cavities are molded into it to represent the ventricles. As in a human heart, the left ventricle (LV) has a conical shape while the right ventricle (RV) has a triangular shape, and they both meet at the extremity of the heart model - the apex. The heart model is $\sim$ 7 cm long, $\sim$ [8 - 8.5] cm large, and $\sim$ 7 cm thick. It is made of Ecoflex (Smooth-On, Macungie, PA, USA) silicon. To mimic the asymmetric behavior of the ventricles in a human heart, the silicon used for each of them in the heart model has a different shore hardness: 00-10 for the left ventricle and 00-50 for the right ventricle (and the rest of the heart model). Additionally, the wall separating the two ventricles, the interventricular septum, is reinforced by a 3D printed PLA plate to reduce its motion and the one of the apex.

The rigid section *(2)* assembles together different elements of the cardiac phantom and creates a closed system. Therefore, it consists of a main compartment containing the contrast solution *(3)*, and it is connected to the heart model *(1)* and to the piston *(4)*. The heart model is screwed in the front of this compartment to create a direct connection for the contrast solution to flow in the heart model. The piston is, on the other hand, inserted in an aperture in the back of the rigid section to enable its sliding in and out of the main compartment. The contrast solution is an aqueous manganese solution (4 ppm Mn^{2+}, 0.8% propylene glycol, 0.04% paraben, 0.04% benzalkolium chloride) and is added to the rigid section through a filling port. This filling system insures the minimal presence of air in the cardiac phantom while allowing its complete filling. The contrast solution being an incompressible fluid, pushing the piston in the rigid section makes it occupy a part of the volume available in the cardiac phantom, and therefore stress is applied on the heart model. In reaction to the stress applied, the heart model deforms, and respectively, it returns to its rest state, or contracts when the piston is pulled out. This design allows to mimic the hemodynamics in the heart as well as the contraction and the expansion of the heart.
